# Brain Functional Networks Study of Subacute Stroke Patients With Upper Limb Dysfunction After Comprehensive Rehabilitation Including BCI Training

**DOI:** 10.3389/fneur.2019.01419

**Published:** 2020-01-27

**Authors:** Qiong Wu, Zan Yue, Yunxiang Ge, Di Ma, Hang Yin, Hongliang Zhao, Gang Liu, Jing Wang, Weibei Dou, Yu Pan

**Affiliations:** ^1^Department of Rehabilitation Medicine, Beijing Tsinghua Changgung Hospital, School of Clinical Medicine, Tsinghua University, Beijing, China; ^2^Institute of Robotics and Intelligent Systems, School of Mechanical Engineering, Xi'an Jiaotong University, Xi'an, China; ^3^Department of Electronic Engineering, Tsinghua University, Beijing, China; ^4^Department of Radiology, Beijing Tsinghua Changgung Hospital, School of Clinical Medicine, Tsinghua University, Beijing, China; ^5^Beijing National Research Center for Information Science and Technology, Beijing, China

**Keywords:** brain computer interface, resting state functional magnetic resonance imaging, stroke, neural plasticity, functional connectivity

## Abstract

Brain computer interface (BCI)-based training is promising for the treatment of stroke patients with upper limb (UL) paralysis. However, most stroke patients receive comprehensive treatment that not only includes BCI, but also routine training. The purpose of this study was to investigate the topological alterations in brain functional networks following comprehensive treatment, including BCI training, in the subacute stage of stroke. Twenty-five hospitalized subacute stroke patients with moderate to severe UL paralysis were assigned to one of two groups: 4-week comprehensive treatment, including routine and BCI training (BCI group, BG, *n* = 14) and 4-week routine training without BCI support (control group, CG, *n* = 11). Functional UL assessments were performed before and after training, including, Fugl-Meyer Assessment-UL (FMA-UL), Action Research Arm Test (ARAT), and Wolf Motor Function Test (WMFT). Neuroimaging assessment of functional connectivity (FC) in the BG was performed by resting state functional magnetic resonance imaging. After training, as compared with baseline, all clinical assessments (FMA-UL, ARAT, and WMFT) improved significantly (*p* < 0.05) in both groups. Meanwhile, better functional improvements were observed in FMA-UL (*p* < 0.05), ARAT (*p* < 0.05), and WMFT (*p* < 0.05) in the BG. Meanwhile, FC of the BG increased across the whole brain, including the temporal, parietal, and occipital lobes and subcortical regions. More importantly, increased inter-hemispheric FC between the somatosensory association cortex and putamen was strongly positively associated with UL motor function after training. Our findings demonstrate that comprehensive rehabilitation, including BCI training, can enhance UL motor function better than routine training for subacute stroke patients. The reorganization of brain functional networks topology in subacute stroke patients allows for increased coordination between the multi-sensory and motor-related cortex and the extrapyramidal system. Future long-term, longitudinal, controlled neuroimaging studies are needed to assess the effectiveness of BCI training as an approach to promote brain plasticity during the subacute stage of stroke.

## Introduction

Recovery of upper limb (UL) motor function after stroke is a critical step for a patient to recover daily activities. Most stroke survivors have acute-stage UL dysfunction, although recovery is incomplete for many (1). Recovery of full UL function is achieved by only 18% of patients who initially present with severe paresis. Furthermore, about 60% of patients with nonfunctional UL at 1 week post-stroke do not fully recover even after 6 months (2). UL dysfunction significantly limits an individual's participation in both physical and social activities (3).

Motor network reorganization after stroke is time- and activity-dependent (4). Hebbian plasticity describes the phenomenon of coincident activation of pre- and post-synaptic neurons, leading to a reinforcement of synaptic strength, finally resulting in increased and more reliable communication between the activated neurons (5, 6). The potential relevance of this concept in behavioral change is particularly well-illustrated in the context of stroke rehabilitation (7). Assuming that the connection between the peripheral muscles and sensorimotor cortex has been disrupted due to the formation of a cortical or subcortical lesion, concurrent activation of sensory feedback loops, combined with activation of the primary motor cortex, may lead to the reinforcement of previously dormant cortical connections via Hebbian plasticity, thereby supporting functional recovery (8, 9). Therefore, it is necessary to develop therapeutic approaches focused on skill learning to promote plasticity, involving enhanced activity of the motor cortex (10). Brain computer interface (BCI) systems allow the brain signals to provide both physical assistance and recovery following central nervous system injury by providing users with brain state-dependent sensory feedback via functional electrical stimulation, virtual reality environments, or robotic systems (11–14). BCI systems can also be used to detect real-time primary motor cortex activation, i.e., the intention to move. As particularly relevant input to BCI systems, EEG signals have highly accurate temporal resolution, are suitable to clinical environments, and can provide matched sensory stimulation according to specific feedback protocols (15, 16). Hence, BCI systems used for motor neurorehabilitation can induce activity-dependent plasticity in specific areas of the brain by requiring the user to pay close attention during task-oriented training, which activates sensorimotor areas (9, 17, 18).

EEG-based BCI strategies have been recently proposed as a promising stroke neurorehabilitation strategy to treat symptoms, including paralysis, cognitive disorders, and aphasia (19–25). Despite the large heterogeneity in the available literature, there is consensus that BCI-based training can help to improve UL motor function in stroke patients.

This is exemplified in the work undertaken by Ramos-Murguialday (26). As compared to placebo expectancy, where orthosis movements occur randomly, significant improvements following BCI training are suggestive of a clinically relevant change from no activity to some voluntary movement of paretic muscles. The electromyography activity of the paretic UL has been correlated to changes in the laterality index, as assessed by fMRI.

However, previous studies do not take account of clinical significance, nor examine clinical effect by of minimal clinically important differences (MCID). MCID signifies smallest change in an outcome measure and can be detected beyond the measurement error. Jaeschke first defined MCID as being “the smallest difference in score in the domain of interest which patients perceive as beneficial and which would mandate, in the absence of troublesome side effects and excessive cost, a change in the patient's management” (27). It is an objective as well as a statistical attribute. Patients who experience an estimated MCID score are more likely to experience a meaningful improvement in disability level than those who do not (28). Researches involved BCI would have been more constructive to clinicians if more attention was paid to MCID.

In addition, another pathway to verify the effectiveness of BCI is to correlate clinical scores with function monitoring. Resting state (rs)-fMRI is used to identify the connectivity traits within the brain that are presumed to be related to neuronal cooperation (29). Many studies have utilized rs-fMRI to measure the activity, spatial extent, and integrity of common measures of FC, such as the default mode network and the sensorimotor network (12, 19, 30–32). Increasing numbers of studies have investigated changes in FC that correlate with motor improvements following BCI training.

In 2013, Várkuti analyzed longitudinal data to examine individual gains in long-term clinical improvements related to FC and demonstrated that increased FC of the supplementary motor area, motor cortex, visuospatial system, and cerebellum was correlated with improved UL function. In other words, changes in FC may be predictive of motor improvement. The authors recommend that future training attempts should focus on directly inducing these beneficial changes (19). One advantage of this study was analysis of the cerebellum, which is often ignored. However, since significant voxels were identified across two groups, the predictors of functional gains in motor function from FC change maps might only represent adaptive processes occurring in the recovering brain, rather than BCI-specific changes.

Young described whole brain network changes correlated with motor recovery following BCI and suggested that the average motor network FC seeded in the thalamus (mainly involving the precuneus, cingulate, paracentral lobule, cerebellum, and superior and middle frontal gyri) was increased mid-therapy and post-therapy relative to baseline. The correlations between FC and behavioral outcomes indicate that both adaptive and maladaptive changes may develop with BCI training (30). However, the study failed to draw a distinction between general increases in FC and non-motor-related FC, which may reveal other neuro-modulatory components of BCI training.

Additionally, machine learning classification was applied to identify the stages of BCI training most beneficial for stroke rehabilitation. Researchers found that regions beside the motor network, such as FCs in fronto-parietal task control, the default mode network, and the subcortical and visual networks, showed similar changes after BCI training. Both strengthening and weakening of FCs were found to be involved in motor and non-motor regions. This study provided new evidence to support the potential clinical utility of BCI training, which not only benefits motor recovery, but also facilitates recovery of other brain functions (32). Furthermore, the study highlighted how machine learning can provide useful information by correlating neuro-function changes (i.e., rs-fMRI, EEG) to behavioral changes (i.e., Action Research Arm Test, Nine-Hole Peg Test, and Barthel Index). They also found that FCs related to the bilateral primary motor area were correlated to behavioral outcomes and clinical variables (33).

BCI-based training can be considered a type of motor learning to modify neuronal activities through sustained feedback and reward. Studies have identified feedback and reward as important contributors to neurorehabilitation (34–37). However, the relationship between BCI training and feedback/reward-related regions of the brain has not been extensively investigated; thus, the efficacy and mechanisms of BCI-based training remain unclear, such as the effects on subacute stroke patients, alterations to sensorimotor area-related networks, and precise relationships with UL function.

Studies have confirmed the clinical benefits of BCI training and brain functional plasticity of UL function in chronic stroke patients. However, in a real world study, most stroke patients engage in rehabilitation with multiform treatments in the subacute stage. However, changes to neural networks in the subacute stage are unclear. As an exploratory study of long-term, controlled research, the aim of the present study was to identify topological alterations in brain functional networks following comprehensive treatment, including BCI training, in subacute stroke patients.

Based on previous studies, we hypothesized that (a) after comprehensive treatments, including BCI training, patients with subacute stroke would develop regional and network topological alterations involving typical hand-related motor regions, as well as sensory/atypical regions; and (b) that these alterations in neural activities would correlate to clinical UL motor function scores.

## Materials and Methods

### Ethical Approval

The study protocol was approved by the Ethics Committee of Beijing Tsinghua Changgung Hospital (Beijing, China) and conducted in accordance with the tenets of the Declaration of Helsinki (approval no. 18172-0-02). All patients provided written informed consent prior to study participation. This study is registered at http://www.chictr.org.cn under the study identifier ChiCTR1900022128.

### Subjects

The study cohort consisted of 25 subacute stroke patients who were recruited from the Department of Physical Medicine and Rehabilitation of Beijing Tsinghua Changgung Hospital (Beijing, China). Each patient underwent a full neurological examination to exclude any accompanying neurological disorders considered as exclusion criteria.

#### Inclusion Criteria

Patients considered for study inclusion met all of the following criteria: (1) age, 18–75 years; (2) sufficient cognition to follow simple instructions and understand the purpose of the study (Mini Mental State Examination, MMSE score >21); (3) hemiparesis resulting from a unilateral brain lesion, as confirmed by MRI, with a time since stroke (TSS) of 1–6 months prior to study enrollment; (4) moderate-to-severe UL paralysis, as determined by a Brunnstrom score ≤ IV; and (5) Modified Ashworth Scale (MAS) score <3.

#### Exclusion Criteria

The exclusion criteria were as follows: (1) severe hand spasticity (MAS score ≥ 3); (2) open wound or deformity of the affected UL; (3) visual field deficit; (4) severe cognitive deficit or receptive aphasia; (5) heavy medication affecting the central nervous system; (6) concomitant serious illness; (7) unilateral spatial neglect; (8) severe dystonia and/or involuntary movements; (9) other neurological disorders, such as severe epilepsy; and (10) participation in another brain stimulation project, such as transcranial direct current stimulation, transcranial magnetic stimulation, or deep brain stimulation, during the training period.

### Baseline Assessment

Baseline clinical scoring included Fugl-Meyer Assessment of the ULs (FMA-UL) (38), the Wolf Motor Function Test (WMFT), and the Action Research Arm Test (ARAT) (39). This study was a randomized control trial of indications employed in previous pilot studies to evaluate the effectiveness of novel rehabilitative interventions (40). The patients were enrolled sequentially and assigned to either the BG or the CG with the use of a pre-designed random number sequence list. Appropriate adjustments were made to balance scores of the most important covariates (i.e., baseline Brunnstrom score, age, sex, TSS, affected hemisphere, type of the lesion, and lesion location). The appointed therapist in charge of the clinical assessment was blinded to the mode of training received by the patients throughout the study period.

### Comprehensive Rehabilitation

All patients received standard medical care and rehabilitation for 4 weeks, which consisted of routine physiotherapy and occupational therapy focused on rehabilitation of arm and hand movements used in daily activities, such as grasping a toothpaste tube, eating, reaching, and grasping while sitting and standing. Each treatment session lasted 2 h in the CG and 1 h in the BG per day, 5 days per week.

On the base of routine training, a BCI training system was developed in the BG, as shown in **Figure 2**. EEG signals were recorded using eight dry electrodes, and then amplified (g.LADYbird, g.Tec Medical Engineering GmbH, Schiedlberg, Austria) and computer processed. A video was projected onto a screen to guide the patient in order to complete each training task. An exoskeleton hand was used to assist the paretic hand in grasping/opening exercises, based on the results of the mu suppression algorithm that was calculated from the EEG signals (41). The system also displayed a mu suppression score on the screen to provide real-time feedback. The mu suppression score provides information about the degree of motor innervation, allowing the patient to adjust in order to achieve higher scores.

EEG signals were referenced to a unilateral earlobe and grounded at the other earlobe. The signal from eight active electrodes was sampled at 256 Hz. EEG signals were also processed in real-time by the amplifier using a band-pass filter (2–60 Hz) and a notch filter (48–52 Hz) to remove artifacts and power line interference, respectively. The EEG electrodes were placed over the central area according to the International 10–20 system (FC3, FC4, C3, C4, CP3, CP4, C1, C2). EEG signals from the C3 and C4 electrodes were used for BCI control. Furthermore, some of the sites related to motor function were used for offline analyses (left hemisphere: FC3, C3, and CP3; right hemisphere: FC4, C4, and CP4). FC3/FC4 covered over the premotor cortex, while C3/C4 covered over the primary motor cortex. CP3/CP4 corresponded to the supramarginal gyrus, which is part of the somatosensory association cortex. These electrodes covered the majority of the sensorimotor cortex.

To compute the mu suppression, the EEG data from C3 to C4 were converted to the frequency domain by a Fourier transform algorithm with a Hanning window covering the EEG data during the video period of the paradigm. The mean power of the mu band (8–13 Hz) for the selected electrode was calculated. Mu suppression reflects an event-related desynchronization of the EEG caused by an increase in neural activity (42). The mu suppression score was calculated according to the following equation (43): where Mu Supp is the Mu suppression score, Mutast is the mu power of the EEG during the motor imagery (MI) task state, and Mu_rest_ is mu power of EEG during the resting state.

$$\begin{array}{l} \text{Mu}supp\;=\;-\frac{Mup_{task}-Mup_{rest}}{Mup_{rest}}*100 \end{array}$$

### BCI Training and Paradigms

**Figure 1:**
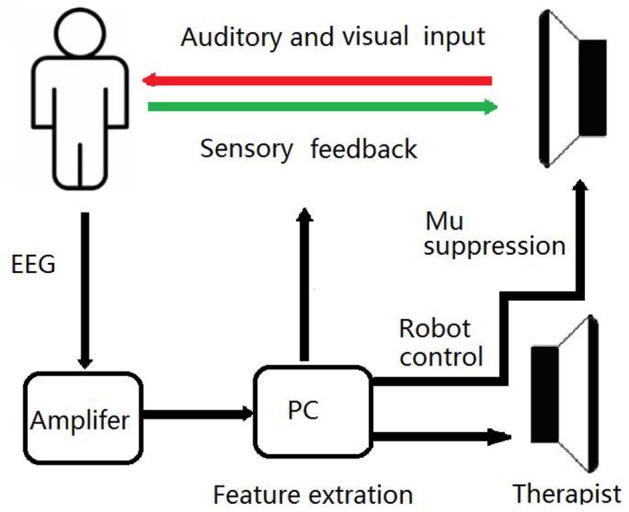
Schematic Diagram of the BCI Training System. During BCI training sessions, patients imagine the movement of affected UL to desynchronize the sensorimotor rhythm. If the mu suppression score was below the threshold, the exoskeleton hand would move.

Patients in the BG received a total of 20 BCI training sessions, lasing for 1 h per day, 5 days per week, for 4 weeks. During the BCI training sessions, patients were instructed to imagine the movement of their affected UL in order to desynchronize sensorimotor rhythm and then to imagine grasping or releasing a cup with the affected hand, after an image-inverted video taken prior of the unaffected hand (Figure 1). The mu suppression score was calculated based on EEG signals during the video clip. An exoskeleton hand provided support to assist the patient with the completion of the hand grasping/opening task during the following 3 s. If the mu suppression score was continuously below the calculated threshold in the motor intention classification area, the exoskeleton hand would move. During each session, the trial was repeated 100 times, and video of the grasping and opening hand was shown alternately at random. Patients were allowed to rest for 1 min after every 10 trials. Patients were instructed to avoid blinking, coughing, chewing, and any other head and body movements.

### Functional Magnetic Resonance Imaging

All fMRI data of the BG were acquired using a GE 3.0T MR scanners (Discovery™ MR750; GE Healthcare Life Sciences, Chicago, IL, USA) before and after training. Participants were scanned in the supine position using a standard 32-channel head-coil. fMRI parameters for rs Blood Oxygen Level Dependent (BOLD) images were an “Ax-BOLD rest” series using a gradient echo planar-imaging sequence, with the following parameters: repetition time = 2,000 ms, echo time = 30 ms, flip angle = 90°, pixel space = 3.5 mm^2^, slice thickness = 3.5 mm, spacing between slices = 4 mm, acquisition matrix = [64, 0, 0, 64] (equivalent to an in-plane resolution of 64 × 64), reconstruction diameter = 224 mm, 34 axial slices, and 240 temporal positions. T1-weighted images (T1) were a “Sag 3D T1BRAVO” series, with repetition time = 8.21 ms, echo time = 3.18 ms, flip angle = 8°, vocal space = 1 mm^3^, spacing between slices = 1 mm, acquisition matrix = [0, 256, 256, 0] (equivalent to 256 axial slices and 256 coronal slices). The sagittal slice number depended on the head size of each patient, and ranged from 156 to 174 mm. The reconstruction diameter was 256 mm.

### FC Analysis

Neuroimaging assessment of FC of the BG was performed by rs-fMRI in three steps: preprocessing, brain network construction, and network feature analysis.

Step 1 was rs BOLD signal preprocessing, which was performed using DPARSFA version 3.2 (http://www.rfmri.org/DPARSF). The first 10 temporal positions of data were discarded to familiarize the patient with the scanning environment. For all remaining temporal positional data. Slice timing correction was performed by phase shifting. The reference slice was set to the slice acquired at the middle time point. Then, head motion was corrected, followed by normalization to the Montreal Neurological Institute (MNI) space with 3 mm isotropic pixel resampling with the direct use of the EPI template. Preprocessing in MNI space included smoothing the data with 4 mm full width at half maximum, while removing the linear trend of the time course, and nuisance covariance regression with head motion, white matter, cerebral fluid, and the whole-brain global signal. Finally, the signal was temporally filtered at a frequency of 0.01–0.08 Hz using an ideal rectangular filter.

**Figure 2:**
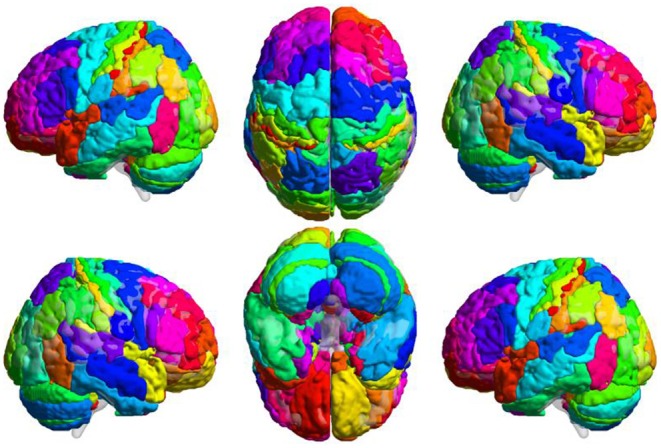
Combined Brain Atlas of the Brodmann Atlas and AAL. Brain atlas was constructed by appending the cerebellum mapping in AAL to the Brodmann atlas. Each colored area presents a functional node in the brain network. This illustration was visualized using BrainNet Viewer (https://www.nitrc.org/projects/bnv/).

Step 2 was brain network construction. We adopted a brain atlas combined with the Brodmann atlas and AAL. This atlas was constructed by appending the cerebellum mapping in AAL (areas 91–116) to the Brodmann atlas (Figure 2). Each area was considered a node in the brain network. At each temporal position, within each area, the average BOLD signal was assigned to the node as the signal intensity. The correlation coefficient between each pair of nodes was calculated as the FC between two regions.

Stroke-related damage to brain tissues may lead to issues during fMRI processing and FC measurements (44), including registration errors and signal disruption. We smoothed data during preprocessing and applied a brain atlas to define the nodes. Smoothing and signal averaging mitigated slight displacement of registration, gave that one region typically contains hundreds- thousands of voxels and smoothing and averaging could blur boundaries. One main issue related to signal disruption is hemodynamic lags. In our work, the patients were in the subacute stage, and according to Siegel, the prevalence of patients showing substantial hemodynamic lags decreases as TSS increases (44). Besides, FC alterations induced by hemodynamic lags following stroke could be taken as a feature of stroke patients, thus it is meaningful to investigate how this feature changes after rehabilitation treatment.

Step 3 was network feature analysis. To investigate network alterations of the BG, seed-based inter-regional correlation analysis was performed. Connections that increased after treatment were identified. The FC was correlated with clinical scores.

### Regions of Interest (ROIs)

ROIs were positioned at the main sensory and motor related cortices that were the source of EEG signals, including the bilateral primary somatosensory cortices (BA1, BA2, BA3), primary motor cortex (BA4), somatosensory association cortex (BA5), premotor cortex (BA6), and superior parietal lobule (BA7). FC changes between the ROIs relative to the whole brain were investigated. Furthermore, the internal relationships between clinical changes and functional reorganization of patients in the BG were explored.

### Outcome Measures

#### Primary Outcome Measures

FC between regions and the whole brain of the BG was the major outcome measure used to detect functional reorganization.

#### Secondary Outcome Measures

The secondary outcomes in this study included the following data of both groups. Demographic data including age and TSS were considered minor (secondary) measures. Clinical score including FMA-UL, ARAT, WMFT before and after training was also secondary measures used to assess changes in UL motor function.

### Statistical Analysis

**Figure 3:**
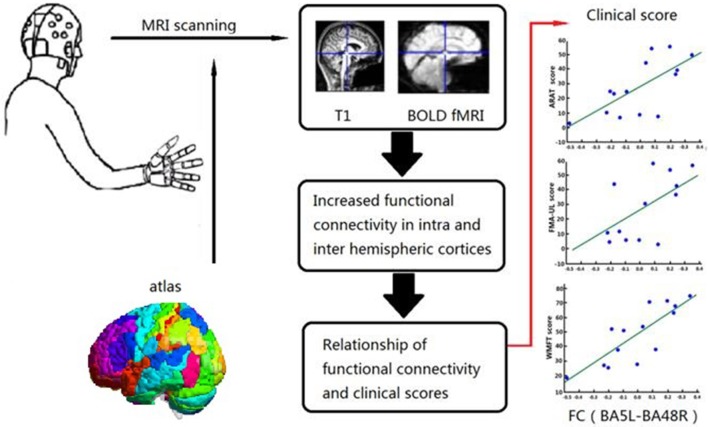
Relationship between Clinical Scores and Functional Alterations. Graphical abstract of this study, patients were assigned to one of BG and CG. Functional assessments of both groups and FC in the BG were performed before and after 4 weeks training. Relationship of FC and clinical scores were analyzed in BG.

All fMRI data were analyzed using NumPy 1.12.1 (http://www.numpy.org) and Scipy 0.19.0 (http://www.scipy.org) software. All demographic and clinical data were analyzed using IBM SPSS Statistics for Windows, version 20.0. (IBM Corporation, Armonk, NY, USA). Normally distributed data are expressed as the mean ± standard deviation. Intergroup comparisons were made using the two-tailed unpaired *t*-test, while intra group comparisons were made using the two-tailed paired *t*-test. Non-normally distributed data are expressed as the median and quartile. The Wilcoxon ranked sum test was used for intra group comparisons and the Mann–Whitney *U*-test for inter group comparisons. The chi-square test was used to identify differences in rates among the groups. Spearman's rank correlation was calculated to assess the relationship between clinical score ranking and corresponding FC of the BG. A probability (*p*) value <0.05 was considered statistically significant for all tests. The entire procedure is shown in Figure 3.

## Results

### Demographics

All of the patients completed training without adverse effects. Tables 1, 2 reported the clinical feature for all patients (pre and post, respectively). Prior to training, the two groups were statistically homogeneous, as there were no significant demographic differences in age (two-tailed unpaired *t*-test, *p* = 0.617), sex (chi-square test, *p* = 0.332) or affected hand (chi-square test, *p* = 0.561). Similarly, there were no significant differences in TSS (Wilcoxon signed-rank test, *p* = 0. 887), lesion type (chi-square test, *p* = 0.332), lesion location (chi-square test, *p* = 0.669) and cognitive impairments (two-tailed unpaired *t*-test, *p* = 0.430). Also, patients in the two groups had similar levels of baseline clinical scores including FMA-UL (two-tailed unpaired *t*-test, *p* = 0.256), ARAT (Wilcoxon rank-sum test, *p* = 0.057), and WMFT (two-tailed unpaired *t*-test, *p* = 0.294).

**Table 1:** Demographic and clinical characters.

**Characteristics**	**BG(*n* = 14)**	**CG(*n* = 11)**	***t*/Z/χ^2^**	***P***
Age (years)	62.93 ± 10.56	64.82 ± 7.22	−0.507^c^	0.617
Sex (male: female)	9:5	9:2	0.939^a^	0.332
Affected hand (right: left)	8:6	6:5	0.337^a^	0.561
TSS (month)	2.11 ± 0.30	2.00(1.50, 3.00)	−0.142^b^	0.887
Type(hemo/isch)	3:11	3:8	0.943^a^	0.332
MMSE score	24.29 ± 2.70	25.18 ± 2.86	−0.803^c^	0.430
FMA -UL score	18.43 ± 2.65	14.09 ± 2.51	1.164^c^	0.256
ARAT score	9.50(3.00, 23.25)	1.00(0.00, 10.00)	−1.900^b^	0.057
WMFT score	30.07 ± 3.38	25.09 ± 2.96	1.074^c^	0.294

*hemo, hemorrhagic stroke; isch, ischemic stroke; TSS, time since stroke; UL-FMA, Upper-Limb Fugl-Meyer Assessment; ARAT, Action Research Arm Test; WMFT, Wolf Motor Function Test*.

a *Chi-square test*;

b *Mann–Whitney U test*;

c *two-tailed unpaired t-test*.

**Table 2:** Lesion maps of patients.

**Affected Vessel**	**Region**	**BG** **(*n* = 14)**	**CG** **(*n* = 11)**
Middle cerebral artery	Basal ganglia	2	2
	Basal ganglia, PLIC	1	1
	Lateral ventricle	1	1
	Basal ganglia, lateral ventricle	3	1
	Thalamus	1	1
	Subtotal	8	6
Posterior circulation	Pons, brainstem	4	2
Internal carotid artery	Frontal lobe, parietal lobe and temporal lobe	2	3
χ^2^	0.804^a^	*P*	0.669

PLIC, posterior limb of internal capsule;

a *Chi-square test of different affected vessel between two groups*.

### Clinical Outcome Measures

Clinical changes after training were observed, with increased scores of FMA-UL, ARAT, and WMFT of both groups, indicating improved UL motor function (Table 3). The intra group differences of both groups after training were statistically significant in all clinical assessments (FMA-UL_BG_, two-tailed paired *t*-test, *p* = 0.000; ARAT_BG_, Wilcoxon signed-rank test, *p* = 0.001; WMFT_BG_, two-tailed paired *t*-test, *p* = 0.000; FMA-UL_CG_, two-tailed paired *t*-test, *p* = 0.008; ARAT_CG_, Wilcoxon signed-rank test, *p* = 0.011; WMFT_CG_, Wilcoxon signed-rank test, *p* = 0.008).

**Table 3:** Results of clinical scores.

		**Pre**	**Post**	**Δ**
FMA-UL	BG	18.43 ± 2.645	35.357 ± 4.255	16.93 ± 2.560
	CG	14.09 ± 2.513	28.071 ± 4.832	8.36 ± 2.116
	**Intra group of BG**	**Intra group of CG**	**Inter group pre**	**Inter group post**
t/*Z*-value	−6.612^d^	−2.673^d^	1.164^c^	−2.549^c^
*p*/Sig. value	0.000^*^	0.008^*^	0.256	0.011^*^
	**Intra group of BG**	**Intra group of CG**	**Inter group pre**	**Inter group post**
ARAT	BG	9.50 (3.00, 23.25)	28.07 ± 4.83	8.50 (4.75, 24.00)
	CG	1.00 (0.00,10.00)	4.00 (3.00,24.00)	4.00 (0.00, 4.00)
	**Intra group of BG**	**Intra group of CG**	**Inter group pre**	**Inter group post**
t/*Z*-value	−3.297^b^	−2.555^b^	−1.900^e^	−2.007^e^
*p*/Sig. value	0.001^*^	0.011^*^	0.057	0.045^*^
	**Intra group of BG**	**Intra group of CG**	**Inter group pre**	**Inter group post**
WMFT	BG	30.07 ± 3.38	47.79 ± 5.00	17.71 ± 3.34
	CG	25.09 ± 2.96	28.00 (18.00, 50.00)	3.00 (1.00, 14.00)
	**Intra group of BG**	**Intra group of CG**	**Inter group pre**	**Inter group post**
t/*Z*-value	−5.298^d^	−2.668^b^	1.074^c^	−2.110^e^
*p*/Sig. value	0.000^*^	0.008^*^	0.294	0.035^*^

b *Mann–Whitney U test*,

c *two-tailed unpaired t-test*;

d *two-tailed paired t-test*;

e *Wilcoxon signed-rank test*;

* *p < 0.05*.

The increased ranges in the BG were ΔFMA-UL: 16.93 ± 2.56, ΔARAT: 8.50 (4.75–24.00) and ΔWMFT: 17.71 ± 3.34. The increased ranges of the CG were ΔFMA-UL: 8.36 ± 2.116, ΔARAT: 4.00 (0.00, 4.00) and ΔWMFT: 3.00 (1.00, 14.00), respectively.

Prior to training, the two groups were statistically homogeneous. No significant clinical differences were found in FMA-UL, ARAT, and WMFT between groups. After training, there were significant inter group differences (FMA-UL_BG−CG_, two-tailed unpaired *t*-test, *p* = 0.011; ARAT_BG−CG_, Mann–Whitney *U*-test, *p* = 0.045; WMFT_BG−CG_, Mann–Whitney *U*-test, *p* = 0.035).

### FC Change

In order to avoid mass data dilution, we only analyzed increased FCs of the BG in this study. The two-tailed paired *t*-test was used to identify significant changes in FC (*p* < 0.05, uncorrected) after treatment. After training, the FCs were found to be increased in the following areas: FC between left BA4 and right BA41, left BA5 and right BA44, and left BA5 and bilateral BA48. BA6 of the right hemisphere was found to be a key brain network node, which connected to left BA37, left BA19, and bilaterally to BA7 (Table 4, Figures 5, 6).

**Table 4:** Functional connectivity between ROI and whole brain network of BG.

**ROI**	**BA**	**Connected Region**	**BA**	**Mean ± SD**	**Mean ± SD**	***t-*value**	***P-*value**
Primary motor cortex	4L	Primary auditory cortex	41R	−0.008 ± 0.212	0.135 ± 0.227	2.317	0.038
Premotor cortex	6R	Superior parietal lobule	7L	−0.177 ± 0.252	0.049 ± 0.214	3.723	0.003
Premotor cortex	6R	Primary somatosensory cortex	2R	0.489 ± 0.133	0.590 ± 0.141	2.542	0.025
Premotor cortex	6R	Primary somatosensory cortex	3R	0.586 ± 0.232	0.697 ± 0.085	2.321	0.037
Premotor cortex	6R	Lateral occipitotemporal cortex	37L	−0.214 ± 0.183	−0.057 ± 0.213	3.092	0.009
Premotor cortex	6R	Superior parietal lobule	7R	0.045 ± 0.228	0.198 ± 0.270	2.774	0.016
Premotor cortex	6R	Associative visual cortex	19L	−0.207 ± 0.227	−0.013 ± 0.287	2.181	0.048
Primary motor cortex	5L	Putamen	48R	−0.128 ± 0.174	−0.006 ± 0.224	2.204	0.046
Primary motor cortex	5L	Putamen	48L	−0.022 ± 0.224	0.088 ± 0.196	2.185	0.048
Primary motor cortex	5R	Pars opercularis	44L	−0.309 ± 0.131	−0.200 ± 0.152	2.682	0.019

*As comparing with the baseline, there were significant increases in FC post-training. L, left hemisphere; R, right hemisphere; BA2, BA3, primary somatosensory cortex; BA4, primary motor cortex; BA5, Somatosensory Association Cortex; BA6, premotor cortex; BA7, superior parietal lobule; BA41, primary auditory cortex; BA37, lateral occipitotemporal cortex; BA19, Associative Visual Cortex; BA44, pars opercularis; BA48, putamen*.

**Figure 4:**
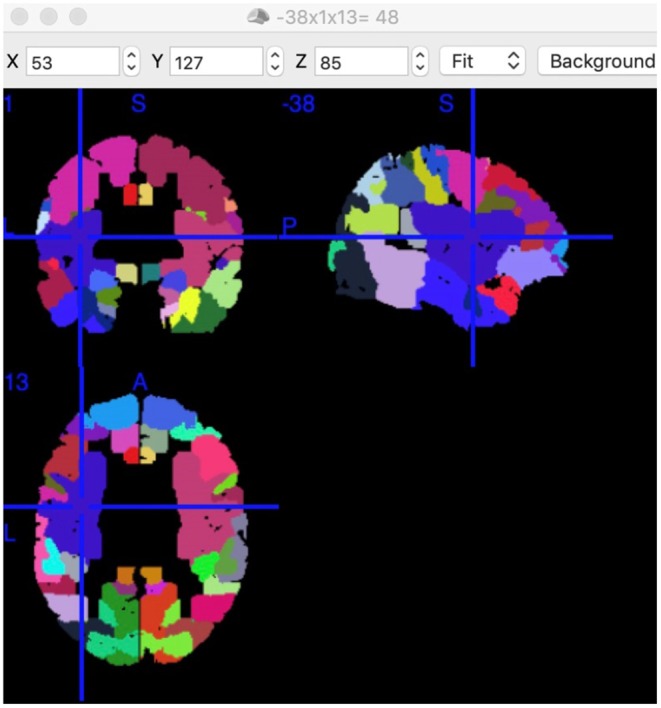
Position of Putamen and BA48. Position of BA48 had a center position on MNI: −38, 1, 13 in this study. Each colored area presents a functional node. This screenshot was captured using MRIcron (https://www.nitrc.org/projects/mricron).

Notably, every Brodmann area contained many voxels with irregular shapes. The region where increased FC connected with left BA5 had a center position on MNI: −38, 1, 13 (Figure 4). According to the Brodmann atlas model, this region corresponded to BA 48 and had a large part to overlap the putamen anatomically.

**Figure 5:**
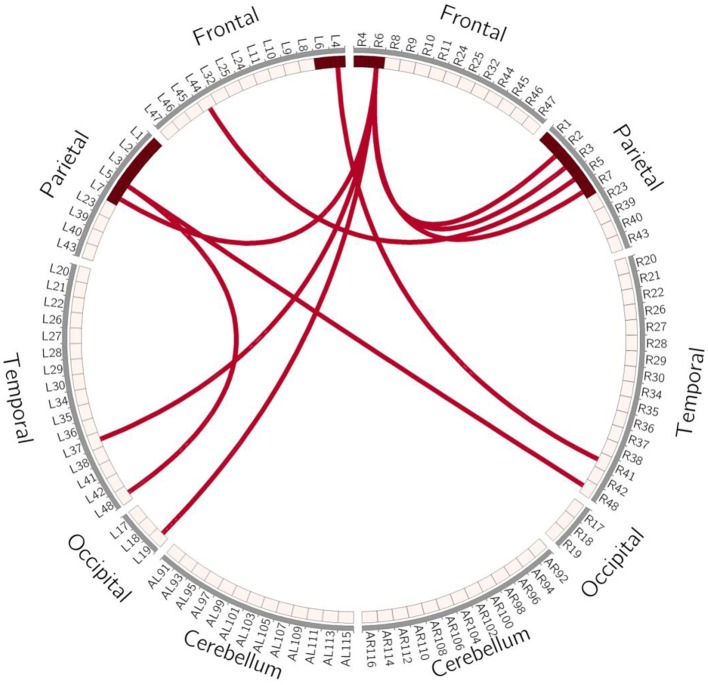
Schematic Diagram of Increased FC in the BG. Red lines represent significantly increased inter- and intra-hemispheric FCs, L, left hemisphere; R, right hemisphere.

**Figure 6:**
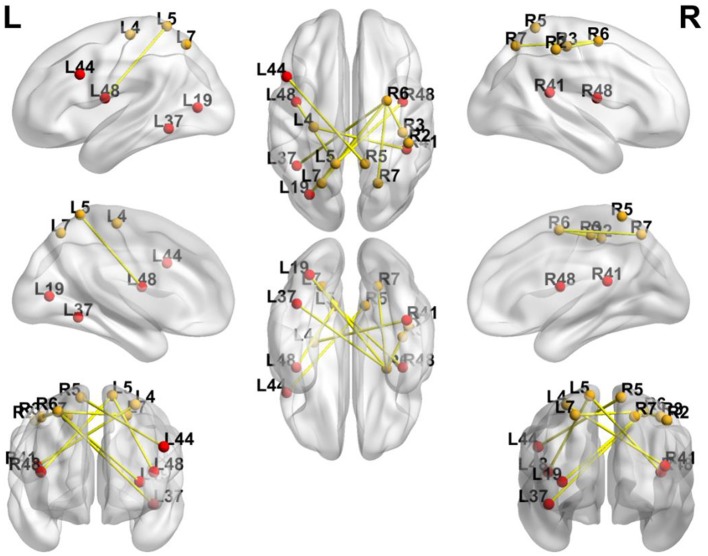
Significance of FC Changes in the BG. Red points and yellow points indicate the significance of FC change in the BG. BA2, BA3, primary somatosensory cortex; BA4, primary motor cortex; BA5, somatosensory association cortex; BA6, Premotor Cortex; BA7, superior parietal lobule; BA41, Primary Auditory Cortex; BA37, lateral occipitotemporal cortex; BA19, associative visual cortex; BA48, putamen; BA44, pars opercularis.

### Correlation Analysis of Increased FCs and Clinical Score

**Figure 7:**
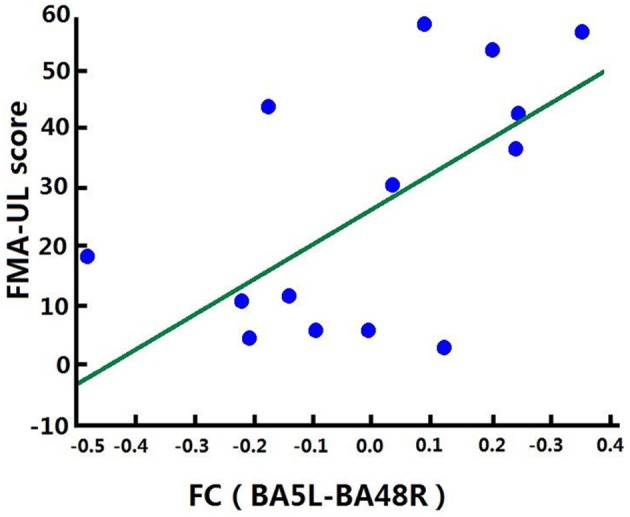
Correlation between FC and FMA-UL_post_ score of the BG. Increases in FC between the left BA5 and the right BA48 were positively correlated with FMA-UL_post_ score after training in the BG. FC (BA5L-BA48R): FC between the left somatosensory association cortex and the right putamen.

**Figure 8:**
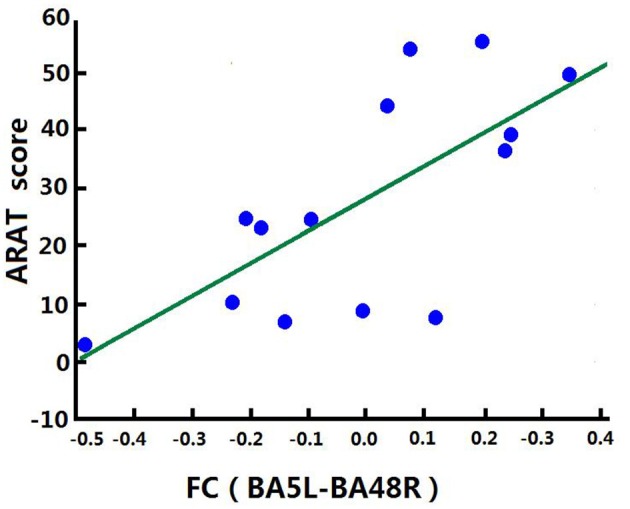
Correlation between FC and ARAT_post_ score of the BG. Increases in FC between the left BA5 and the right BA48 were positively correlated with ARAT_post_ score after training in the BG.

**Figure 9:**
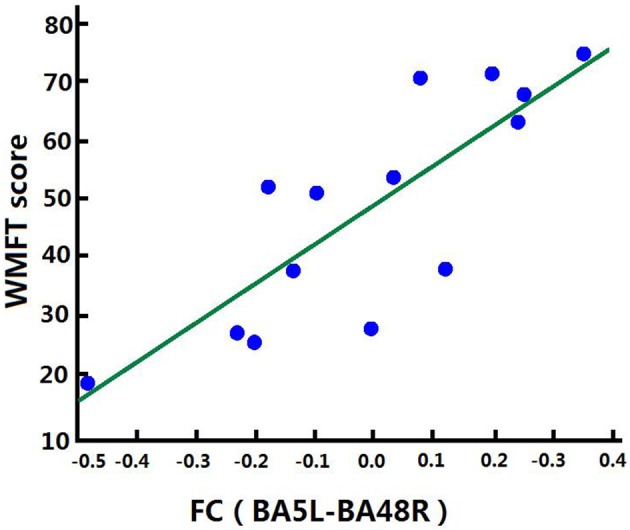
Correlation between FC and WMFT_post_ score of the BG. Increases in FC between the left BA5 and the right BA48 were positively correlated with WMFT_post_ score after training in the BG.

Correlation analysis was performed to assess the relationship between FC and clinical score in the BG (FMA-UL, ARAT, and WMFT scores). After comprehensive rehabilitation, including BCI training, increases in FC between the left BA5 and right BA48 were positively correlated with clinical scores post training: FMA-UL_post_ score (Spearman's rank correlation, *r* = 0.641, *p* = 0.013; Figure 7), ARAT_post_ score (Spearman's rank correlation, *r* = 0.701, *p* = 0.005; Figure 8), and WMFT_post_ score (Spearman's rank correlation, *r* = 0.814, *p* = 0.000; Figure 9).

## Discussion

The results of the present study demonstrate that after comprehensive rehabilitation, including BCI training, there were significant clinical improvements in UL function of subacute stroke patients. The improved clinical scores significantly surpassed routine training.

In addition to comparing with CG, we also focus on MCID. As for patients with subacute stroke, the MCID of FMA-UL is 9–10 (28). There are no MCID results for ARAT and WMFT in the subacute stage of stroke. In this study, the improvement of FMA-UL of BG not only significantly higher than that of CG, but also surpass MCID. Therefore, we concluded that clinical effect of comprehensive rehabilitation including BCI training is better than routine training. It is worth noting that the concept of MCID does not specify study duration. The clinical effect of the routine training may take longer time to manifest. The advantages of BCI training needs further observation.

Similar to the study of (45) of stroke patients with a TSS of 6 weeks to 6 months, comparison of BCI-monitored MI practice and training showed better FMA score in the BG. These results demonstrate the rehabilitative potential of BCI, which contributes to significantly better motor functional outcomes in subacute stroke patients with UL motor impairments.

The definition of “subacute” was limited to within 1–6 months after onset in consideration of the influence of spontaneous recovery (46) and relatively stable blood flow (47). In this stage, patients were able to receive more intensive training, i.e., 2–3 h per day.

Concomitant rs-MRI data and correlation with measures of clinical improvement of the BG suggested possible mechanisms underlying these changes. The inter- and intra-hemispheric FCs between multiple brain regions were significantly enhanced and involved typical motor-related regions, such as the primary and premotor cortices. Moreover, we observed changes to atypical motor-unrelated regions, such as the visuospatial, visual, sensory, and somatosensory regions, as well as the primary auditory cortex. These changes may be expected to arise from the nature of BCI training. However, only FC between the somatosensory association cortex and the putamen were specifically associated with clinical improvements after BCI training. These results suggest that the extrapyramidal system may play an important role in hand control and functional recovery, with the help of sensory input.

MI-based BCI can be viewed as a special form of “motor behavior,” which activates areas associated with the selection of actions and multi-sensory integration, including the premotor cortex, anterior cingulum, and parts of the superior and inferior parietal cortices (48). Our findings suggest similar increased activities among these regions. Among these areas, the premotor cortex is considered a key node, since most increased FCs are connected with it, and plays a role in direct control of certain behaviors, such as planning, as well as spatial and sensory guidance of movement, with neurons show responsiveness to stimulation of tactile, vision, and audition. It also participates in learning processes by associating sensory stimulation with specific movements or learning rules (49).

Our results indicate that the premotor cortex seemed to be crucial for the coordination and concentrate variety of functions. During BCI training, patients were required to concentrate on a video of hand movements using different tools and then to repeat these movements using mental imagery. The close relationships observed between visual and motor system was characteristic of BCI training, and was consistent with known neurofeedback dynamics occurring in the brains of patients following stroke (50). The abundance of visual signals activated the primary visual cortex (BA19), while activation of the lateral occipitotemporal cortex (part of BA37) was likely related to hand-specific visual processing. In addition, the superior parietal lobule, part of BA7, which is involved in locating objects in space and in visuo-motor coordination, serves as a point of convergence between vision and proprioception in order to determine where objects are in relation to parts of the body (51). Sensory input from the UL may also play an important role in BCI training, since training involved continuous movements of the exoskeleton and routine training. Those movements likely led to the increased FC between the primary somatosensory cortex (BA2, BA3) and premotor cortex, as they relate to perception, feedback, and accurate modeling of MI. Comprehensive rehabilitation, including BCI training, can be considered as “enriched environment” training, as it integrates visual, auditory, sensory, and cognitive information simultaneously to promote functional recovery.

Outside of the typical motor-related network, we observed atypical sensory-motor integration after training. BA4, corresponding to the primary motor cortex, is the primary region of the motor system, which works together with other systems to execute movements. Previous research indicates that BA4 also plays a key role in the early stages of motor learning and may be involved in the transition from early motor memory to long-term motor memory (52). During BCI training, patients maintained relatively constant accuracy over sessions as the task difficulty gradually increased, which supports the hypothesis that BCI training promotes an adaptive learning process. BA41 is part of the superior temporal gyrus, well-known as the auditory cortex, and is involved in a network of maintaining perceptual representations during memory-based tasks and perceptual decision-making. In an auditory discrimination task using both positive and negative reinforcement, BA41 was found to be not only responsive to reward, but also to avoidance of punishment during feedback presentation (53). In the present study, patients received two auditory signals during training, the first being a pre-warning prior to the onset of movement on the screen, and the second being feedback regarding accuracy after the movement. The auditory stimulus in this context is different from language or music. For musicians, the modulation of auditory-motor networks occurs mainly between the premotor area and the auditory cortex (54). In this study, the auditory stimulus functioned as a form of conduct training. Once familiarized, the patients did not need to distinguish the auditory stimulus, thus it shifted as an auditory signal that assisted in making an executive decision.

Beyond the premotor cortex, there were also FC changes between the somatosensory association cortex (BA5) and extrapyramidal regions. The pars opercularis (BA44) is part of Broca's area, which has non-language related functions, such as the formation of complex hand movements, associative sensorimotor learning, and sensorimotor integration (55). The observed increased FC between the somatosensory association cortex and the pars opercularis may be due to its involvement in perception and sensory feedback in complex hand movements.

In this study, only one increased FC between BA48 and BA5 was related to all clinical assessments after training, indicating the importance of the extra-vertebral system and sensory integration during the recovery of motor function. The extra-vertebral system is another important channel involved in motor control in charge of reward-based learning. BA48 is overlapped with the putamen, in the striatum. The putamen is an important integrative interface between visualization and motor intention during the process of mental rotation, which allows smooth and accurate rotation. Anatomically, the putamen forms a sensory-motor cognitive loop, which is connected to the motor cortices and the somatosensory cortex. Functionally, the putamen has been shown to be involved during the initiation of unskilled movements that require high levels of cognitive control, as well as the automatic processing of well-learned automated hand movements (56). The combination of sensory signals may help to complete the imagined spatial rotation of the hand. More importantly, the accuracy and smoothness of hand movements serve as further positive feedback for functional improvement. Therefore, enhanced FC between BA5 and BA48 can be thought of being related to clinical improvement in UL function after BCI training.

There are several features that distinguish this study from previous reports. First, few previous studies have focused on FC changes of subacute stroke patients who received BCI training. However, during the subacute phase, patients have greater potential than in the chronic phase. This study is critical for further understanding of the neural plasticity mechanisms of motor function recovery, which will improve the effectiveness of present therapies. Second, in our FC analysis, we chose direction-time correlated original values instead of absolute values, because the former more accurately represents functional motor changes after training. Additionally, we analyzed FC by combining a hemispheric Brodmann template and an AAL template for the brain stem and cerebellum, respectively, to allow a more integrated analysis of neural plasticity. Although the cerebellum plays an important role in fast and skilled movements and working memory, there was no increase in FC between the cerebellum and ROIs in this study. On the other hand, in a similar study of subacute and chronic patients, increased FC between the cerebellum and motor cortex was correlated with improved UL function after BCI training (19). The difference in these results may be related to the original values used in this study.

This study had several limitations. First, although we compared clinical improvements of comprehensive rehabilitation, which included BCI and routine training, the characteristics of spontaneous recovery on neuroimaging were not eliminated. Second, a large number of patients had weakened FCs, which may be related to clinical changes. However, the weakened FCs showed significant chaos and heterogeneity. To avoid mass data dilution, only increased FCs assessed in this study. Third, the lesions were located in different hemispheres and corresponding vessels in this study. Since there might be significant differences in the recovery patterns between hemispheres and affected areas, further analyses of lesion position, neuroplasticity and clinical effect are crucial.

In conclusion, this study compared clinical improvements of comprehensive rehabilitation, including BCI training and routine training, and described the region and network topology alterations in subacute stroke patients following BCI training. We found that subacute stroke patients after BCI training not only showed better motor recovery, but also activities in other brain networks, including somatosensory, visual spatial processing, and motor learning. The extra-vertebral system may be involved in the improvement of motor function. Our findings suggest that after comprehensive rehabilitation, including BCI training, there was reorganization of brain functional networks topology in subacute stroke patients, thereby allowing increased coordination between multi-sensory and motor related cortex and the extrapyramidal system. We hope that this paper would give rise to more innovations to tackle the potential pathway of neurorehabilitation intervention. Future long-term, longitudinal, controlled neuroimaging studies are needed to identify the effectiveness of BCI training and approaches to promote brain plasticity in the subacute stage of stroke.

## Data Availability Statement

The datasets generated for this study are available on request to the corresponding author.

## Ethics Statement

The studies involving human participants were reviewed and approved by Ethics Committee of the Beijing Tsinghua Changgung Hospital. The patients/participants provided their written informed consent to participate in this study.

## Author Contributions

QW, YP, WD, and JW designed the study. DM, HY, GL, ZY, and HZ performed experiments. WD and YG analyzed data. QW and ZY wrote the paper. All authors reviewed and approved the final version of the manuscript.

### Conflict of Interest

The authors declare that the research was conducted in the absence of any commercial or financial relationships that could be construed as a potential conflict of interest.

## Abbreviations

BCI: Brain Computer Interface
UL: Upper Limb
FMA-UL: Fugl-Meyer Assessment-UL
ARAT: Action Research Arm Test
WMFT: Wolf Motor Function Test
FC: Functional Connectivity
EEG: Electroencephalography
EMG: Electromyography
fMRI: Functional Magnetic Resonance Imaging
TSS: Time Since Stroke
MAS: Modified Ashworth Scale
tDCs: Transcranial Direct Current Stimulation
TMS: Transcranial Magnetic Stimulation
DBS: Deep Brain Stimulation
BOLD: Blood Oxygen Level Dependent
MNI: Montreal Neurological Institute
FWHM: Full-Width-Half-Maximum
ROI: Regions of Interest
BA: Brodmann Area
MCID: Minimal Clinically Important Differences.
